# A Unified Approach to Two-Dimensional Brinkman-Bénard Convection of Newtonian Liquids in Cylindrical and Rectangular Enclosures

**DOI:** 10.3390/e26010002

**Published:** 2023-12-19

**Authors:** Pradeep G. Siddheshwar, Kanakapura M. Lakshmi, David Laroze

**Affiliations:** 1 Centre for Mathematical Needs, Department of Mathematics, CHRIST (Deemed to Be University), Hosur Road, Bengaluru 560029, India; pg.siddheshwar@christuniversity.in; 2 Department of Mathematics, School of Physical Sciences, Central University of Karnataka, Kalaburagi 585367, India; 3 Instituto de Alta Investigación, Universidad de Tarapacá, Casilla 7D, Arica 1000000, Chile; dlarozen@academicos.uta.cl

**Keywords:** Brinkman–Bénard convection, cylindrical and rectangular geometries, onset of convection

## Abstract

A unified model for the analysis of two-dimensional Brinkman–Bénard/Rayleigh–Bénard/ Darcy–Bénard convection in cylindrical and rectangular enclosures ( CE/RE ) saturated by a Newtonian liquid is presented by adopting the local thermal non-equilibrium ( LTNE ) model for the heat transfer between fluid and solid phases. The actual thermophysical properties of water and porous media are used. The range of permissible values for all the parameters is calculated and used in the analysis. The result of the local thermal equilibrium ( LTE ) model is obtained as a particular case of the LTNE model through the use of asymptotic analyses. The critical value of the Rayleigh number at which the entropy generates in the system is reported in the study. The analytical expression for the number of Bénard cells formed in the system at the onset of convection as a function of the aspect ratio, $S_{o}$ , and parameters appearing in the problem is obtained. For a given value of $S_{o}$ it was found that in comparison with the case of LTE , more number of cells manifest in the case of LTNE . Likewise, smaller cells form in the DBC problem when compared with the corresponding problem of BBC . In the case of RBC , fewer cells form when compared to that in the case of BBC and DBC . The above findings are true in both CE and RE . In other words, the presence of a porous medium results in the production of less entropy in the system, or a more significant number of cells represents the case of less entropy production in the system. For small and finite $S_{o}$ , the appearance of the first cell differs in the CE and RE problems.

## 1. Introduction

Rayleigh–Bénard convection ( RBC ) in a fluid-saturated porous medium has been widely studied in the last few decades due to its wide range of applications in many fields, including thermal insulation systems, geothermal reservoirs, cooling of electronic and thermal equipment, etc. Generally, in nature, two types of porous media are seen, viz., high-porosity (loosely packed) and low-porosity (densely packed) media. Mathematically, loosely packed porous media are modelled using the Brinkman–Forchheimer-extended Darcy/Brinkman model. The densely packed porous medium is modelled using the Darcy model. These models consider the geometrical properties of the medium. Observing the thermal properties of the fluid and the porous medium, one adopts either the local thermal equilibrium ( LTE ) or the local thermal nonequilibrium ( LTNE ) model. When the difference exists in the thermal properties of fluid and solid phases, then the consideration of the LTNE model becomes necessary to analyse the system.

We first present a detailed literature survey concerning RBC in a loosely-packed porous medium (Brinkman–Bénard convection— BBC ) followed by that of a densely-packed porous medium (Darcy–Bénard convection— DBC ) occupying a rectangular geometry and a cylindrical geometry with the LTNE assumption. The articles studying BBC with LTNE [1,2,3,4,5,6,7,8] and DBC with LTNE [9,10,11,12,13,14] in rectangular enclosures ( RE ) are covered below. Phanikumar and Mahajan [1] examined the heat transfer characteristics of natural convection flow in a fluid-saturated metal-foam porous medium heated from below. They modelled the porous medium flow using the Brinkman–Forchheimer-extended Darcy model ( BFED model). They conclude that the LTNE model is a better model to study convection in the fluid-metal-foam porous medium. Postelnicu and Rees [2] performed a linear stability analysis of BBC of a Newtonian liquid using the LTNE model. They found excellent agreement between the asymptotic and the numerical results of heat transfer. Using the LTNE model, Straughan [3] determined the threshold value of convection at which instability occurs by making use of a global nonlinear stability analysis. He showed that the critical value predicted by the nonlinear theory exactly matches the one predicted by the linear theory. Khashan et al. [4] numerically simulated the natural convection problem in fluid-saturated porous cavities, including non-Darcian effects: Brinkman, convecting and Forchheimer terms. The effect of non-Darcian terms is well examined over a wide range of Rayleigh numbers. Postelnicu [5] made a linear stability analysis of DBC with LTNE assumption using single-term and *N*-term Galerkin methods. Using a single-term Galerkin method, an explicit expression is obtained for the Rayleigh number as a function of the wave number and other parameters. The eigenvalue obtained from this has been used as an initial guess value for the Galerkin method. Siddheshwar and Siddabasappa [6] analytically made linear and non-linear stability analyses of BBC using the LTNE model with rigid and free boundary conditions. The results of the Darcy model are obtained as a limiting case. Liu et al. [7] numerically investigated the flow structure and the heat transfer of BBC in a square lattice. They examined the effect of porosity on the flow properties during the transition of flow from BBC to DBC . Siddheshwar et al. [8] analytically studied the BBC problem of four visco-elastic/one Newtonian fluid-saturated media occupying rectangular/cylindrical enclosures. They developed a common Lorenz model for both CE and RE to analyse the dynamics of the system. We next move on to the literature survey concerning DBC – LTNE problems.

Banu and Rees [9] numerically investigated the onset of DBC in a fluid-saturated porous medium using the two-temperature model between fluid and solid phases. The results of the LTE model are recovered by taking the thermal equilibrium limits (asymptotic analyses). Govender and Vadasz [10] performed a linear stability analysis of DBC with Coriolis and gravitational body forces and concluded that the effect of rotation is to stabilise convection even when the system exhibits thermomechanical anisotropy. Postelnicu [11] studied the effect of pressure gradient on the onset of DBC using the Galerkin method and the numerical solver dsolve of Maple. The results of the LTE model are recovered for large values of the scaled interface heat transfer parameter. Bidin and Rees [12] made a weakly non-linear analysis of the classical DBC problem to analyse the effect of the LTNE assumption on different planforms of convection. Secondary instabilities like Eckhaus and zig-zag instabilities were also considered. They showed that the roll planform is a stable planform of convection. Siddheshwar et al. [13] investigated both regular and chaotic convection in a densely-packed porous medium saturated by a Newtonian liquid by considering phase lag effects. A novel decomposition method is used to obtain an extended Vadasz-Lorenz model. They found from the study that the phase-lag effects alter the nature of chaos. Bansal and Suthar [14] analysed the onset of DBC using the LTNE model when the temperature of the two boundaries varies sinusoidally. The matrix differential operator theory is adopted to perform the stability analysis, and the critical Rayleigh number as a function of system parameters is obtained. The problem of RBC in RE is well investigated by many researchers and is now documented in books [15,16,17,18,19,20]. Kita [21] showed in his study the principle of maximum entropy as a general rule to study the stability of a RBC in non-equilibrium steady states. Jia et al. [22] studied the characteristics of RBC systems entropy production using the centre mesh systems and the finite volume method. They found from the study that the Rayleigh number and the aspect ratio play a crucial role in the formation of the Bénard cells pattern in the system. We next present the literature survey of CE .

There is no reported literature on BBC with LTNE , DBC with LTNE and BBC with LTE (except the work of Siddheshwar et al. [8], the study is restricted to unicellular convection) in a cylindrical geometry. Thus, we shall now focus our attention on the literature survey of DBC with LTE approximation in cylindrical enclosures ( CE ) and cylindrical annuli ( CA ) [23,24,25,26,27,28,29,30,31,32]. Zebib [23] analytically studied the onset of DBC in cylindrical enclosures. They found that the asymmetric mode is the preferred mode of convection except in the range of aspect ratio (radius/height) [1.09:1.28] at which the axisymmetric mode of convection occurs. Bau and Torrance [24] examined the onset of DBC in cylindrical annuli with two types of top wall boundary conditions, viz., permeable and impermeable. They showed that the system is more stable in the case of impermeable boundary condition compared to the permeable one. Haugen and Tyvand [25] performed a linear stability analysis of DBC with impermeable and conducting boundary conditions. They showed that the axisymmetric mode of convection is always a preferred one. In their analysis, they predicted the number of cells for some ranges of aspect ratios using the stream function plots. Zhang et al. [26] determined the critical Darcy–Rayleigh number for DBC in a cylindrical enclosure saturated by a visco-elastic fluid. They also determined the preferred mode of convection and found it to be a function of the aspect ratio and visco-elastic parameters. Bringedal et al. [27] examined the onset of DBC in a cylindrical porous annulus with impermeable, heat-conducting or insulating boundary conditions. They found that the effect of the inserted solid cylinder on the onset of convection is more dominant in the case of conducting boundary conditions compared to the insulating one. Kuznetsov and Nield [28] analytically investigated the onset of through-flow convection in a porous cylinder with vertical heterogeneity using a single-term Galerkin method. They found from the study that due to the symmetry of the horizontal boundary condition, the through flow is stabilising. Barletta and Storesletten [29] analysed the onset of convection in a densely packed vertical porous cylinder. The Robin boundary condition on temperature is applied to the horizontal boundaries, and the side boundary is assumed to be thermally insulated. The study shows that the Biot number plays an important role in determining the transition of the aspect ratio from one mode to the other. They also showed that the principle of exchange of stabilities is valid in the study. Kang et al. [30] analysed the onset of convection in a visco-elastic fluid-saturated rotating porous annulus. They examined the effect of the Taylor number and visco-elastic parameters on the onset and the mode of convection. Barletta and Storesletten [31] performed a linear stability analysis on DBC in a porous cylinder with permeable and thermally insulated vertical boundaries. Hence, the side wall is constrained by the temperature and pressure distributions. They first report results on DBC in a circular cylinder and then generalise the study to arbitrary cross-sectional cylinders. Siddheshwar and Lakshmi [32] analytically studied the onset and the heat transport by DBC in cylindrical enclosures and cylindrical annuli. They found from the study that the onset is advanced, and the heat transport is enhanced in the case of a cylindrical annulus compared to a cylindrical enclosure when the same volume is considered in the two geometries. The problem of RBC is one of the limiting cases of the BBC problem. Having completed a literature survey of RBC in CE , we found that [33,34,35,36,37,38,39,40] have considered the problem.

From the literature survey on Newtonian fluid occupying a porous medium, we found that the following aspects have not yet been investigated:

- Analytical study of BBC in CE with LTE/LTNE .
- Analytical study of RBC in CE .
- Analytical study of DBC in CE with LTNE .

In addition to the above-mentioned open problems, there are no reported results on aspects concerning the dependence of the number of cells and cell size on the aspect ratio, thermophysical properties and geometrical properties of the porous medium. Most importantly, explicit expression connecting cell size with the parameters representing these properties does not seem to be there. It is the endeavour of this paper to find such an expression and gain insight into the nature of cells that form. Also, our paper intends to report a unified analysis covering all the above-mentioned unconsidered problems in cylindrical and rectangular enclosures.

## 2. Mathematical Formulation

Analytical study of two-dimensional Rayleigh–Bénard convection in a fluid-saturated sparsely-packed porous medium confined in cylindrical/rectangular enclosures is considered for investigation. The considered porous medium is assumed to be isotropic and made up of spherically shaped porous solids. The lower and upper plates of the enclosures are assumed to be thin and maintained at temperatures $T_{0}+\Delta T$ and $T_{0}$ , respectively, with ΔT>0 , and are separated by a distance *d*. The horizontal dimension of the enclosures is taken as *b*. The chosen coordinate system has *s* and *y* axes, respectively, along the radial/horizontal and vertical directions.

(1) $$\frac{\partial u}{\partial s}+\frac{\delta }{\delta (1-s)+s}\frac{u}{s}+\frac{\partial v}{\partial y}=0,$$

(2) $$\frac{\rho_{f}}{\phi }\frac{\partial \vec{Q}*}{\partial t}=-\nabla^{*}P+\mu_{e}\nabla^{{*}^{2}}\vec{Q}*-\frac{\mu_{f}}{K}\vec{Q}*+\left[\rho_{f}-\left(\rho_{f}\beta \right)\left(T_{f}-T_{0}\right)\right]\vec{g},$$

(3) $$\left(\rho_{f}c_{f}\right)\left[\phi \frac{\partial T_{f}}{\partial t}+\left(\vec{Q}*\cdot \nabla^{*}\right)T_{f}\right]=\phi k_{f}\nabla^{{*}^{2}}T_{f}+h\left(T_{s}-T_{f}\right),$$

(4) $$\left(\rho_{s}c_{s}\right)\left(1-\phi \right)\frac{\partial T_{s}}{\partial t}=k_{s}\left(1-\phi \right)\nabla^{{*}^{2}}T_{s}-h\left(T_{s}-T_{f}\right),$$

where $\vec{Q}*=u\left(s,\;y,\;t\right)\hat{e_{s}}+v\left(s,\;y,\;t\right)\hat{e_{y}}$ is the two-dimensional velocity vector, δ is the curvature parameter (artificially introduced) which takes two values 0 and 1 corresponding to the problems of cylindrical and rectangular enclosures, respectively. The quantities $\phi =\frac{\mathrm{Volume}\;\mathrm{of}\;\mathrm{pores}}{\mathrm{Volume}\;\mathrm{of}\;(\mathrm{pores}\;+\;\mathrm{porous}\;\mathrm{matrix})}$ is the porosity, $K=\frac{\phi^{3}d_{s}^{2}}{180{\left(1-\phi \right)}^{2}},\;d_{s},\;\rho ,\;c,\;\mu ,\;t,P,$ β,g,T,k and *h* are, respectively, permeability, diameter of the solid spheres (porous medium), density, specific heat at constant pressure, dynamic viscosity, time, pressure, thermal expansion coefficient, acceleration due to gravity, temperature, thermal conductivity and heat transfer coefficient. The operator $\nabla^{*}=\hat{e_{s}}\frac{\partial }{\partial s}+\hat{e_{y}}\frac{\partial }{\partial y}$ is the two-dimensional gradient operator. The subscripts f,s,e,and0 , respectively, represent the quantities pertaining to fluid, solid, effective quantities (in terms of fluid and solid phases’ values) and reference value. The reference value is taken at $T=T_{0}$ .

The pressure term in Equation (2) will now be eliminated by operating curl twice, and then on simplifying, we obtain the equation for the *y*-component of velocity as

(5) $$\frac{\rho_{f}}{\phi }\frac{\partial }{\partial t}\left(\nabla^{{*}^{2}}v\right)=\mu_{e}\nabla^{{*}^{4}}v-\frac{\mu_{f}}{K}\nabla^{{*}^{2}}v+\left(\rho_{f}\beta \right)g\nabla_{s}^{2}T_{f},$$

where $\nabla^{{*}^{2}}=\nabla_{s}^{2}+\frac{\partial^{2}}{\partial y^{2}}$ is the Laplacian operator and $\nabla_{s}^{2}=\frac{\partial^{2}}{\partial s^{2}}+\frac{\delta }{s}\frac{\partial }{\partial s}$ is the horizontal Laplacian operator.

The dimensional quantities present in Equations (1)–(4) are non-dimensionalised using the following quantities:

(6) $$\begin{array}{cc} \; & \tau =\frac{\chi_{f}}{d^{2}}t,\;\left(S,\;Y\right)=\left(\frac{s}{b},\;\frac{y}{d}\right),\;\left(U,\;V\right)=\frac{d}{\phi \chi_{f}}\left(u,\;v\right),\;\left(\Theta ,\;\Phi \right)=\frac{\left(T_{f},\;T_{s}\right)}{\Delta T}, \end{array}$$

where $\chi =\frac{k}{\rho c}$ is the thermal diffusivity.

On non-dimensionalising Equations (1)–(4) using Equation (6), we obtain:

(7) $$\frac{1}{S_{o}}\frac{\partial U}{\partial S}+\frac{\delta }{S_{o}\left[\delta (1-S)+S\right]}\frac{U}{S}+\frac{\partial V}{\partial Y}=0,$$

(8) $$\frac{1}{Pr}\frac{\partial }{\partial \tau }\left(\nabla^{2}V\right)=\Lambda \nabla^{4}V-\sigma^{2}\nabla^{2}V+Ra\nabla_{S}^{2}\Theta ,$$

(9) $$\frac{\partial \Theta }{\partial \tau }=\nabla^{2}\Theta -\left(\vec{Q}\cdot \nabla \right)\Theta +H\left(\Phi -\Theta \right),$$

(10) $$\alpha \;\frac{\partial \Phi }{\partial \tau }=\nabla^{2}\Phi -H\gamma \left(\Phi -\Theta \right).$$

The non-dimensional parameters present in Equations (7)–(10) are defined as follows: (Brinkman [41])

(11) $$\left.\begin{array}{cc} Pr=\frac{\phi \mu_{f}}{\rho_{f}\chi_{f}}\;\left(\begin{array}{c} \mathrm{porosity}-\mathrm{modified}\\ \mathrm{Prandtl}\;\mathrm{number} \end{array}\right),\;\;\; & \alpha =\frac{\chi_{f}}{\chi_{s}}\;\begin{array}{c} (\mathrm{thermal}\;\mathrm{diffusivity}\\ \;\mathrm{ratio}) \end{array},\\ H=\frac{hd^{2}}{\phi k_{f}}\;\left(\begin{array}{c} \mathrm{scaled}\;\mathrm{inter}-\mathrm{phase}\\ \mathrm{heat}\;\mathrm{transfer}\;\mathrm{coefficient} \end{array}\right), & \Lambda =\frac{\mu_{e}}{\mu_{f}}\;\left(\mathrm{Brinkman}\;\mathrm{number}\right),\\ \gamma =\frac{\phi k_{f}}{\left(1-\phi \right)k_{s}}\;\left(\begin{array}{c} \mathrm{porosity}-\mathrm{weighted}\\ \mathrm{thermal}\;\mathrm{conductivity}\;\mathrm{ratio} \end{array}\right), & \sigma^{2}=\frac{d^{2}}{K}\;\left(\mathrm{porous}\;\mathrm{parameter}\right),\\ Ra=\frac{\rho_{f}\beta g\Delta Td^{3}}{\phi \mu_{f}\chi_{f}}\;\left(\begin{array}{c} \mathrm{porosity}-\mathrm{modified}\\ \mathrm{thermal}\;\mathrm{Rayleigh}\;\mathrm{number} \end{array}\right), & S_{o}=\frac{b}{d}\;(\mathrm{aspect}\;\mathrm{ratio}),\\ \mu_{e}=\frac{\mu_{f}}{\phi^{2.5}}\;. & \end{array}\right.$$

The operators appearing in Equations (7)–(10) are:

(12) $$\left.\begin{array}{cccc} \; & \nabla_{S}^{2}=\frac{1}{S_{o}^{2}}\frac{\partial^{2}}{\partial S^{2}}+\frac{1}{S_{o}^{2}}\frac{\delta }{S}\frac{\partial }{\partial S}, & \nabla^{2} & =\frac{1}{S_{o}^{2}}\frac{\partial^{2}}{\partial S^{2}}+\frac{1}{S_{o}^{2}}\frac{\delta }{S}\frac{\partial }{\partial S}+\frac{\partial^{2}}{\partial Y^{2}}. \end{array}\right.$$

At the quiescent basic state, the system is in an entropy equilibrium condition due to the assumption that the destabilising temperature variations are compensated by the stabilising effect (caused by the viscosity) of the fluid-saturated porous medium. Hence, we assume that the heat is transported by conduction alone, and there is local thermal equilibrium ( LTE ) between the phases. Thus, we take

(13) $$\vec{Q}=0,\;\;\Theta =\Theta_{b}\left(Y\right),\;\;\Phi =\Phi_{b}\left(Y\right)\;\;\mathrm{and}\;\;\Theta_{b}=\Phi_{b}.$$

With the Equation (13), the governing Equations (7)–(10) reduce to:

(14) $$\frac{d^{2}\Theta_{b}}{dY^{2}}=0,\;\;\;\frac{d^{2}\Phi_{b}}{dY^{2}}=0.$$

The basic state Equation (14) is solved subject to the following conditions:

(15) $$\begin{array}{c} \Theta =\Theta_{0}+1,\;\;\Phi =\Phi_{0}+1\;\;\mathrm{at}\;\;Y=0\;\;\mathrm{and}\;\;0<S<1,\\ \Theta =\Theta_{0},\;\;\Phi =\Phi_{0}\;\;\mathrm{at}\;\;Y=1\;\;\mathrm{and}\;\;0<S<1 \end{array}$$

and the resultant solution is:

(16) $$\Theta_{b}=1-Y,\;\;\;\Phi_{b}=1-Y.$$

Now, to check the stability of the system, we superimpose a small perturbation on the quiescent basic state, which generates entropy in the system, as:

(17) $$\vec{Q}=\vec{Q^{\prime }},\;\;\Theta =\Theta_{b}+\Theta^{\prime },\;\;\Phi =\Phi_{b}+\Phi^{\prime },$$

where prime denotes a perturbed quantity. Using Equation (17) in the governing Equations (7)–(10) and then using Equation (16) in the resulting equations, we obtain:

(18) $$\frac{1}{S_{o}}\frac{\partial U^{\prime }}{\partial S}+\frac{\delta }{S_{o}\left[\delta (1-S)+S\right]}\frac{U^{\prime }}{S}+\frac{\partial V^{\prime }}{\partial Y}=0,$$

(19) $$\frac{1}{Pr}\frac{\partial }{\partial \tau }\left(\nabla^{2}V^{\prime }\right)=-\sigma^{2}\nabla^{2}V^{\prime }+\Lambda \nabla^{4}V^{\prime }+Ra\;\nabla_{S}^{2}\Theta^{\prime },$$

(20) $$\frac{\partial \Theta^{\prime }}{\partial \tau }=\nabla^{2}\Theta^{\prime }-\left(\vec{q^{\prime }}\cdot \nabla \right)\Theta^{\prime }+V^{\prime }+H\left(\Phi^{\prime }-\Theta^{\prime }\right),$$

(21) $$\alpha \frac{\partial \Phi^{\prime }}{\partial \tau }=\nabla^{2}\Phi^{\prime }-H\gamma \left(\Phi^{\prime }-\Theta^{\prime }\right).$$

Further on, in Equations (18)–(21), we neglect primes for simplicity and solve them subject to the following boundary conditions:

Impermeable, stress-free and isothermal horizontal boundaries

(22) $$V=0,\;\mu_{e}\left[\frac{\partial U}{\partial Y}+\frac{\partial V}{\partial S}\right]=0,\;\Theta =0,\;\Phi =0\;\;\mathrm{on}\;Y=0,\;1\;\mathrm{and}\;0<S<1.$$

Impermeable, stress-free and adiabatic vertical boundaries

(23) $$V=0,\;\mu_{e}\left[\frac{\partial V}{\partial S}+\frac{\partial U}{\partial Y}\right]=0,\;\frac{\partial \Theta }{\partial S}=0,\;\frac{\partial \Phi }{\partial S}=0\;\mathrm{on}\;S=0,\;1\;\mathrm{and}\;0<Y<1.$$

On simplifying Equations (22) and (23) using the continuity Equation (18), we obtain

(24) $$\left.\begin{array}{cc} V=\frac{\partial^{2}V}{\partial Y^{2}}=0,\;\;\Theta =0,\;\;\Phi =0\;\;\mathrm{on}\;\;Y=0,\;1\;\;\; & \mathrm{and}\;0<S<1,\\ U=\nabla_{S}^{2}U=0,\;\;\frac{\partial \Theta }{\partial S}=0,\;\;\frac{\partial \Phi }{\partial S}=0\;\;\mathrm{on}\;\;S=0,\;1\;\;\; & \mathrm{and}\;0<Y<1, \end{array}\right.$$

where the boundary S=0 in the case of CE is a pseudo-boundary.

Having obtained the nondimensional form of governing equations and boundary conditions, we now present the linear stability analysis to determine the critical Rayleigh number at which convection occurs.

## 3. Linear Stability Analysis under the Assumption of the Principle of Exchange of Stabilities

On linearising Equations (19)–(21) and considering the steady state, we obtain:

(25) $$\begin{array}{c} -\sigma^{2}\nabla^{2}V+\Lambda \nabla^{4}V+Ra\nabla_{S}^{2}\Theta =0, \end{array}$$

(26) $$\begin{array}{c} \nabla^{2}\Theta +V+H\left(\Phi -\Theta \right)=0, \end{array}$$

(27) $$\begin{array}{c} \nabla^{2}\Phi -H\gamma \left(\Phi -\Theta \right)=0. \end{array}$$

The Y− dependent part of the variable separable eigenfunctions corresponding to the periodically appearing roll planform (velocity) and for isothermal conditions (fluid and solid temperatures) are of the form sin[πY] . Thus, we take

(28) $$V\left(S,\;Y\right)=V^{*}\left(S\right)\sin\left[\pi Y\right],\;\Theta \left(S,\;Y\right)=\Theta^{*}\left(S\right)\sin\left[\pi Y\right],\;\Phi \left(S,\;Y\right)=\Phi^{*}\left(S\right)\sin\left[\pi Y\right].$$

The solution (28) satisfies *Y*-boundary conditions given in Equation (24). In view of this, for the purpose of calculation, we may now write $\nabla_{S}^{2}-\pi^{2}$ for $\nabla^{2}$ . With this, Equations (25)–(27) now become:

(29) $$\begin{array}{c} \Lambda \nabla_{S}^{4}V^{*}-\left(\sigma^{2}+2\pi^{2}\Lambda \right)\nabla_{S}^{2}V^{*}+\pi^{2}\left(\sigma^{2}+\pi^{2}\Lambda \right)V^{*}+Ra\nabla_{S}^{2}\Theta^{*}=0, \end{array}$$

(30) $$\begin{array}{c} \left(\nabla_{S}^{2}-\pi^{2}\right)\Theta^{*}+V^{*}+H\left(\Phi^{*}-\Theta^{*}\right)=0, \end{array}$$

(31) $$\begin{array}{c} \left(\nabla_{S}^{2}-\pi^{2}\right)\Phi^{*}-H\gamma \left(\Phi^{*}-\Theta^{*}\right)=0. \end{array}$$

Decoupling of $V^{*},\;\Theta^{*}$ and $\Phi^{*}$ from Equations (29)–(31) and the resulting equations are unified as follows:

(32) $$\begin{array}{c} \nabla_{S}^{8}\varpi^{*}-\left[q_{1}+\frac{q_{2}}{\Lambda }+2\pi^{2}\right]\nabla_{S}^{6}\varpi^{*}+\left[\left(\pi^{2}+q_{1}\right)\left(2\pi^{2}+\frac{q_{2}}{\Lambda }\right)+\frac{\sigma^{2}\pi^{2}-Ra}{\Lambda }\right]\nabla_{S}^{4}\varpi^{*}\\ +\left[\frac{Ra}{\Lambda }q_{3}-\pi^{4}\left(q_{1}+\frac{q_{2}}{\Lambda }\right)-2\pi^{2}q_{1}\frac{q_{2}}{\Lambda }\right]\nabla_{S}^{2}\varpi^{*}+\frac{\pi^{4}}{\Lambda }q_{1}q_{2}\varpi^{*}=0, \end{array}$$

where

(33) $$\varpi^{*}={\left[V^{*}\;\Theta^{*}\;\Phi^{*}\right]}^{T},\;\;q_{1}=\pi^{2}+H\left(1+\gamma \right),\;\;q_{2}=\sigma^{2}+\pi^{2}\Lambda ,\;\;q_{3}=\pi^{2}+H\gamma .$$

To solve Equation (32), we need 8 boundary conditions with respect to *S* on each of $V^{*},\;\Theta^{*}$ and $\Phi^{*}$ . Equation (24) gives us only two boundary conditions on Θ and Φ and four boundary conditions on *U*. But we need boundary conditions only on *V*, and hence we convert *U* boundary conditions present in Equation (24) into *V* boundary conditions using Equation (18). This procedure gives us:

(34) $$\frac{\partial V^{*}}{\partial S}=\frac{\partial }{\partial S}\left(\nabla_{S}^{2}V^{*}\right)=0\;\;\mathrm{on}\;\;S=0,\;1\;\;\;\mathrm{and}\;0<Y<1.$$

At this point, we are short of four and six boundary conditions on $V^{*}$ and $\left(\;\Theta^{*},\;\Phi^{*}\right)$ , respectively, with respect to *S*. The additional boundary conditions required are obtained using Equations (29)–(31) and the available boundary conditions. The required boundary conditions on adding new ones on $V^{*},\;\Theta^{*}$ and $\Phi^{*}$ are:

(35) $$\frac{\partial \varpi^{*}}{\partial S}=\frac{\partial }{\partial S}\left(\nabla_{S}^{2}\varpi^{*}\right)=\frac{\partial }{\partial S}\left(\nabla_{S}^{4}\varpi^{*}\right)=\frac{\partial }{\partial S}\left(\nabla_{S}^{6}\varpi^{*}\right)=0\;\;\mathrm{on}\;\;S=0,\;1\;\;\mathrm{and}\;\;0<Y<1.$$

Let us now factorise Equation (32) as follows:

(36) $$\left(\nabla_{S}^{2}+m^{2}\right)\left(\nabla_{S}^{2}+n^{2}\right)\left(\nabla_{S}^{2}+p^{2}\right)\left(\nabla_{S}^{2}+q^{2}\right)\varpi^{*}=0,$$

where $m^{2},\;n^{2},\;p^{2}$ and $q^{2}$ are to be determined. On multiplying together the four factors in Equation (36), we obtain

(37) $$\begin{array}{c} \nabla_{S}^{8}\varpi^{*}+\left(m^{2}+n^{2}+p^{2}+q^{2}\right)\nabla_{S}^{6}\varpi^{*}+\left(m^{2}n^{2}+m^{2}p^{2}+m^{2}q^{2}+n^{2}p^{2}+n^{2}q^{2}+p^{2}q^{2}\right)\nabla_{S}^{4}\varpi^{*}\\ +\left(m^{2}n^{2}p^{2}+m^{2}n^{2}q^{2}+n^{2}p^{2}q^{2}+m^{2}p^{2}q^{2}\right)\nabla_{S}^{2}\varpi^{*}+m^{2}n^{2}p^{2}q^{2}\varpi^{*}=0. \end{array}$$

Now on comparing Equations (32) and (37), we obtain the following relations connecting $m^{2},\;n^{2},\;p^{2}$ and $q^{2}$ :

(38) $$\begin{array}{cc} & m^{2}+n^{2}+p^{2}+q^{2}=-\left[q_{1}+\frac{q_{2}}{\Lambda }+2\pi^{2}\right], \end{array}$$

(39) $$\begin{array}{cc} & m^{2}\left(n^{2}+p^{2}+q^{2}\right)+n^{2}p^{2}+n^{2}q^{2}+p^{2}q^{2}=\left[\left(\pi^{2}+q_{1}\right)\left(2\pi^{2}+\frac{q_{2}}{\Lambda }\right)+\frac{\sigma^{2}\pi^{2}-Ra}{\Lambda }\right], \end{array}$$

(40) $$\begin{array}{cc} & m^{2}n^{2}p^{2}+m^{2}n^{2}q^{2}+n^{2}p^{2}q^{2}+m^{2}p^{2}q^{2}=\left[\frac{Ra}{\Lambda }q_{3}-\pi^{4}\left(q_{1}+\frac{q_{2}}{\Lambda }\right)-2\pi^{2}q_{1}\frac{q_{2}}{\Lambda }\right], \end{array}$$

(41) $$\begin{array}{cc} & m^{2}n^{2}p^{2}q^{2}=\frac{\pi^{4}}{\Lambda }\left(q_{1}q_{2}-2Ra\right). \end{array}$$

In the above equations, with the intention of retaining only $m^{2}$ we rewrite Equations (38), (39) and (41) as

(42) $$\begin{array}{c} n^{2}+p^{2}+q^{2}=-\left[q_{1}+\frac{q_{2}}{\Lambda }+2\pi^{2}\right]-m^{2}, \end{array}$$

(43) $$\begin{array}{c} n^{2}p^{2}+n^{2}q^{2}+p^{2}q^{2}=\left[\left(\pi^{2}+q_{1}\right)\left(2\pi^{2}+\frac{q_{2}}{\Lambda }\right)+\frac{\sigma^{2}\pi^{2}-Ra}{\Lambda }\right]-m^{2}\left(n^{2}+p^{2}+q^{2}\right), \end{array}$$

(44) $$\begin{array}{c} n^{2}p^{2}q^{2}=\frac{\pi^{4}}{\Lambda }\left(q_{1}q_{2}-2Ra\right)/m^{2}. \end{array}$$

We next simplify Equation (40) as:

(45) $$\begin{array}{c} m^{2}\left(n^{2}p^{2}+n^{2}q^{2}+p^{2}q^{2}\right)+n^{2}p^{2}q^{2}=\left[\frac{Ra}{\Lambda }q_{3}-\pi^{4}\left(q_{1}+\frac{q_{2}}{\Lambda }\right)-2\pi^{2}q_{1}\frac{q_{2}}{\Lambda }\right]. \end{array}$$

On using Equations (42)–(44) in the Equation (45), we obtain

(46) $$\begin{array}{c} {\left(m^{2}\right)}^{4}+\left[q_{1}+\frac{q_{2}}{\Lambda }+2\pi^{2}\right]{\left(m^{2}\right)}^{3}+\left[\left(\pi^{2}+q_{1}\right)\left(2\pi^{2}+\frac{q_{2}}{\Lambda }\right)+\frac{\sigma^{2}\pi^{2}-Ra}{\Lambda }\right]{\left(m^{2}\right)}^{2}\\ -\left[\frac{Ra}{\Lambda }q_{3}-\pi^{4}\left(q_{1}+\frac{q_{2}}{\Lambda }\right)-2\pi^{2}q_{1}\frac{q_{2}}{\Lambda }\right]m^{2}+\frac{\pi^{4}}{\Lambda }\left(q_{1}q_{2}-2Ra\right)=0. \end{array}$$

The Equation (46) may be used to solve for m , but it involves the eigenvalue Ra . In view of this we rearrange Equation (46) to obtain the expression for Ra in the form:

(47) $$Ra=\frac{{\left(m^{2}+\pi^{2}\right)}^{2}}{m^{2}}\left[\Lambda \left(m^{2}+\pi^{2}\right)+\sigma^{2}\right]\left[1+\frac{H}{m^{2}+\pi^{2}+H\gamma }\right].$$

The parameter Ra is an eigenvalue which characterises the production of entropy in the system once it crosses its threshold value. We call it a critical Rayleigh number, $Ra_{c}$ .

At this point, we shift our attention to obtaining the solution of V,Θ and Φ of Equations (25)–(27). In what follows, we shall obtain the solution for the Equation (36) for $\varpi^{*}$ . We hence choose the Helmholtz Equation:

(48) $$\left(\nabla_{S}^{2}+m^{2}\right)\varpi^{*}=0.$$

The solution of the Helmholtz Equation in (48) is:

(49) $$\left.\begin{array}{c} \varpi^{*}=AS^{\frac{1-\delta }{2}}J_{\frac{\delta -1}{2}}\left[mS_{o}S\right], \end{array}\right.$$

where $A={\left[A_{0}\;B_{0}\;C_{0}\right]}^{T}$ is the matrix of infinitesimal amplitudes of convection, $J_{\frac{\delta -1}{2}}\left[mS_{o}S\right]$ is the Bessel function of the first kind and of order $\frac{\delta -1}{2}$ . The solution in Equation (49) is used in Equation (28) to obtain the complete solution of $\varpi ={\left[V\;\Theta \;\Phi \right]}^{T}$ in the form:

(50) $$\;\begin{array}{c} \varpi =AS^{\frac{1-\delta }{2}}J_{\frac{\delta -1}{2}}\left[mS_{o}S\right]\sin\left[\pi Y\right]. \end{array}$$

Equation (50) satisfy the boundary condition in Equation (35), provided:

(51) $$J_{\frac{1+\delta }{2}}\left[mS_{o}\right]=0.$$

From the above proceeding it becomes evident that the critical m , viz., $m_{c}$ , is not just the result of the minimisation of Ra with respect to m . It also involves the constraint condition (51). Thus, we have on hand a constraint minimisation problem involving Equations (47) and (51). To simplify the constraint minimisation problem further, we make the substitution $a=mS_{o}$ in Equations (47) and (51). With this substitution, Equations (47) and (51) now take the form:

(52) $$Ra=\frac{{\left(a^{2}/S_{o}^{2}+\pi^{2}\right)}^{2}}{a^{2}/S_{o}^{2}}\left[\Lambda \left(a^{2}/S_{o}^{2}+\pi^{2}\right)+\sigma^{2}\right]\left[1+\frac{H}{a^{2}/S_{o}^{2}+\pi^{2}+H\gamma }\right],$$

(53) $$J_{\frac{1+\delta }{2}}\left[a\right]=0.$$

There are infinitely more solutions for a that satisfy the condition (53). Among these values of a , a particular value which minimises the Rayleigh number is the required critical value of a , namely $a_{c}$ .

After having evolved the right procedure for obtaining the critical values of a and thereby $m_{c}$ and $Ra_{c}$ in the case of the unified problem, we next discuss the results obtained in the study.

## 4. Results and Discussion

The present article reports various problems of axisymmetric CE and two-dimensional RE in a unified way. Various problems unified into one problem shall be explained in brief now. More details on the limiting cases shall be given later on in the section. Brinkman–Bénard convection ( BBC ) of Newtonian liquids in axisymmetric CE and two-dimensional RE is studied analytically(by artificially introducing the curvature parameter) using the two-temperature model adopted for the LTNE situation. When the thermal properties of the two phases (fluid and solid) are quite different, then the LTNE assumption is required to be considered. In such a case, the two-temperature model will have to be used to study the thermo-fluid dynamics. This situation is all the more true when an enormous temperature is observed in the system. However, when the temperature involved is not that high, then the temperatures of the two phases do not differ much when the values of thermophysical quantities are nearly the same. This situation is termed as LTE . One can mathematically obtain the results of the LTE model from those of the LTNE model by making asymptotic analyses [2,6,9]. In the present analysis, we have considered a loosely packed porous medium. By neglecting factors that characterise the porous medium, we can study the results of the clear fluid case. With the intention of obtaining the results of a densely-packed porous medium, we may consider small permeability and, hence very large values of the porous parameter.

**Figure 1:**
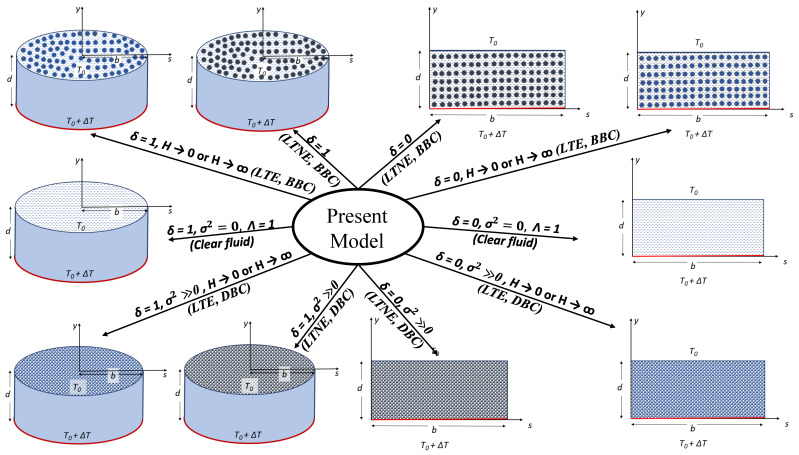
Schematic representation of the present model.

From the above, it is clear that several problems can be unified into a single problem (see Figure 1). In what follows, we present the following problems (limiting cases) in essential detail:

- Two-dimensional BBC problems in CE and RE with LTNE .
- Two-dimensional DBC problems in CE and RE with LTNE .
- Two-dimensional RBC problems in CE and RE .
- Two-dimensional BBC problems in CE and RE with LTE .
- Two-dimensional DBC problems in CE and RE with LTE .

As a result of the unified handling, we shall be dealing with ten limiting cases as one and the same is shown in Figure 1.

In the present paper, the material of the porous medium is such that its thermophysical properties are much different from those of the fluid occupying it. Hence, of relevance to this paper is the LTNE situation only. However, for academic completeness, we consider the LTE situation too and arrive at some results. The very general formulation adopted here involves a loosely packed porous medium that allows us to handle both the no porous medium and the densely packed porous medium cases.

To make the results have a practical value, we have made use of the actual thermophysical properties of the fluid and of the porous matrix. The details are provided in the next subsection.

### 4.1. Thermophysical Properties and the Parameters’ Values concerning a Fluid-Saturated Porous Medium

The fluid and porous media considered in the analysis are water, glass fibre ( GF ), sand, glass balls ( GB ) and aluminium foam ( AF ) porous medium, and their thermophysical properties are present in the articles [42,43]. Among the four porous media considered, GF has high porosity; hence, we use that for a specific investigation concerning BBC . The other three porous media have low porosity; hence, we use them in the study of DBC . Using the thermophysical properties mentioned in Table 1, we shall now calculate various parameters, Pr,α,Λ,γ,H and $\sigma^{2}$ appearing in Section 2.

The parameter $Pr=\frac{\phi \mu_{f}}{\rho_{f}\chi_{f}}$ is the porosity-modified Prandtl number. Since Pr is a function of ϕ , it is found to be different for GF(ϕ=0.88) and other porous media ( ϕ=0.5 ). The permissible value of Pr for different water-saturated porous media is calculated, and the same is recorded in the first column of Table 1. The diffusivity ratio, $\alpha =\frac{\chi_{f}}{\chi_{s}}$ , is calculated and its values are recorded in the second column of Table 1. The values of $\gamma =\frac{\phi k_{f}}{\left(1-\phi \right)k_{s}}$ are computed for four different fluid-saturated porous media and are recorded in the third column of Table 1. The parameter, $\Lambda =\frac{\mu_{e}}{\mu_{f}}$ , is the ratio of effective viscosity to the viscosity of the fluid. The effective viscosity is calculated using the phenomenological law, $\mu_{e}=\frac{\mu_{f}}{\phi^{2.5}}$ and Λ values are documented in the fourth column of Table 1.

The four parameters discussed above are dependent only on the thermal properties of the fluid and porous media and do not depend on the height of the enclosures. The parameters $H=\frac{hd^{2}}{\phi k_{f}}$ and $\sigma^{2}=\frac{d^{2}}{K}$ are clearly dependent on the height of the enclosure, where $K=\frac{\phi^{3}d_{s}^{2}}{180{\left(1-\phi \right)}^{2}}$ is the permeability of porous media. In the paper, we choose two different heights ( d=0.02 m, 0.03 m) of enclosures and the obtained parameters’ values that depend on *d* are tabulated in Table 2. In the experimental work of Liang et al. [33], they mention that asymmetric convection is observed if the height of the enclosure is greater than 0.03 m. Since our study concerns axisymmetric convection, we have chosen d≤0.03 m.

To find the permissible value for the parameter *H*, it is required to find the heat transfer coefficient, *h*. Kuwahara et al. [44] reported a numerical study to find a correlation for *h* as a function of Pr , Reynold’s number, $Re=\frac{\rho_{e}d\;V}{\mu_{e}}$ , and ϕ , where $\rho_{e}=\phi \rho_{f}+\left(1-\phi \right)\rho_{s}$ is the effective density. The fluid flow velocity, *V*, in the cases of high-porosity and low-porosity media, is chosen as 0.01 m/s and 0.001 m/s, respectively. The expression of *h* from Kuwahara et al. [44] is given by

(54) $$h=\frac{k_{f}}{d_{s}}\left[\left(1+\frac{4(1-\phi )}{\phi }\right)+\frac{1}{2}\sqrt{\left(1-\phi \right)}Re^{0.6}Pr^{1/3}\right].$$

Also, *h* depends on the diameter of the solid spheres, $d_{s}$ . Here, we have chosen the value of $d_{s}$ in the range of [0.005:0.02] m and [0.001:0.005] m, respectively, for the cases of loosely and densely-packed porous media. The notation [a:b] implies that the quantity takes the least value of *a* and the maximum value of *b*. Now, using Equation (54), we found the permissible range for *H* for the considered porous media and the same is tabulated in Table 2. The parameters K and $\sigma^{2}$ are found for the above-mentioned range of values of $d_{s}$ , and the same are also tabulated in Table 2.

Having discussed the thermophysical properties and having estimated the non-dimensional parameters, we now consider the limiting cases: RBC and DBC and obtain the expression of the Rayleigh number in the two cases.

### 4.2. Expressions of the Rayleigh Number in the Cases of RBC and DBC

We first consider the case of RBC . In the case of no porous medium, the value of ϕ is 1 and, hence, $\mu_{e}=\mu_{f}$ . For this value of ϕ and $\mu_{e}$ , K is infinite and hence the parameters $\sigma^{2}$ and Λ take the values 0 and 1, respectively. Now the Rayleigh number expression (52) in the case of a clear fluid takes the form:

(55) $$Ra=\frac{{\left(a^{2}/S_{o}^{2}+\pi^{2}\right)}^{3}}{a^{2}/S_{o}^{2}}.$$

We next consider the DBC problem. From the recorded values of $\sigma^{2}$ in Table 2, one can observe that the value of $\sigma^{2}$ is very large in the case of densely-packed porous media (sand, GB and AF ) compared to the finite value of $\sigma^{2}$ in the case of a loosely-packed porous medium( GF ). When $\sigma^{2}\gg 1$ , then clearly $\Lambda \left(a^{2}/S_{o}^{2}+\pi^{2}\right)\ll \sigma^{2}$ and hence the second term in $\sigma^{2}+\Lambda \left(a^{2}/S_{o}^{2}+\pi^{2}\right)$ may be neglected.

The Equation (52) now takes the form:

(56) $$Ra_{D}=\frac{{\left(a^{2}/S_{o}^{2}+\pi^{2}\right)}^{2}}{a^{2}/S_{o}^{2}}\left[1+\frac{H}{a^{2}/S_{o}^{2}+\pi^{2}+H\gamma }\right],$$

where $Ra_{D}=\frac{Ra}{\sigma^{2}}$ is the Darcy–Rayleigh number.

After obtaining Ra expressions in the cases of BBC , RBC and DBC , in the next subsection, we shall consider asymptotic analyses to obtain the results of the LTE from the LTNE .

### 4.3. Asymptotic Analyses: LTNE to LTE

The results of LTE can be extracted from those of LTNE using asymptotic analyses. This is possible in four different ways, as illustrated by [2,6,9]. The routes to LTE from LTNE are:

(57) $$\left.\begin{array}{ccc} \; & \left(i\right) & H\to 0,\\ \; & \left(\mathrm{ii}\right) & H\to \infty ,\\ \; & \left(\mathrm{iii}\right) & \gamma \to \infty ,\\ \; & \left(\mathrm{iv}\right) & H\gamma \to \infty . \end{array}\right.$$

The result of extensive asymptotic analyses carried out by Postelnicu and Rees [2] concerns the cases of BBC and DBC . The expression of the Rayleigh number of the LTE case that emerges from an asymptotic analysis in the cases of BBC and DBC is documented in Table 3.

The expression of the Rayleigh number for BBC and DBC clearly indicate that Ra is a function of a and $S_{o}$ . In the following section, we determine, through the constraint condition, the value of a that minimises Ra for a given value of $S_{o}$ .

### 4.4. General Solution of the Constraint Condition: $J_{\frac{1+\delta }{2}}\left[a\right]$ = 0

We find the roots of the constraint condition in the cases of five problems, each of CE and RE , that we have unified.

For δ=0 , the constraint takes the form:

(58) sin[a]=0.

The roots of Equation (58) are:

(59) a=nπ,n=1,2,⋯.

For δ=1 , the constraint simplifies to the form:

(60) $$J_{1}\left[a\right]=0.$$

The Equation (60) yields discrete values as the solution. Let us name them as $a_{0},\;a_{1},\;a_{2},$ $a_{3},\cdots$ , where $a_{0}<a_{1}<a_{2}<a_{3}<\cdots$ . The first seven values of a (for δ=0 and δ=1 ) are reported in Table 4. From the above, it is clear that a has an explicit expression for RE whereas a has discrete values only in CE , and an explicit expression does not occur as in the case of RE . In attempting to construct an explicit expression for a in CE , we considered 100 discrete values of a , viz., $a_{0},\;a_{1},\;a_{2},\cdots ,a_{99}$ . Making a close observation of these values, we found that $|a_{n+1}-a_{n}|\approx 3.1416\left(\approx \pi \right)$ for n≥2 . Making use of this idea to find an explicit expression for a we tried out different linear interpolation functions: $a_{0}+n\pi ,\;a_{1}+\left(n-1\right)\pi ,\;a_{2}+\left(n-2\right)\pi ,\cdots a_{5}+\left(n-5\right)\pi$ , and found $a_{2}+\left(n-2\right)\pi$ to be the line of best fit. Hence, we chose the following:

(61) $$a=a_{2}+\left(n-2\right)\pi ,\;\;n=1,\;2,\;3\cdots .$$

In order to find a unified expression for a in five problems each of CE and RE , we now write Equations (59) and (61) in unified form as:

(62) $$a=(a_{2}-2\pi )\delta +n\pi ,\;\;n=1,\;2,\cdots .$$

We need to mention here that as yet, it is not clear as to what *n* and $m=\frac{a}{S_{o}}$ represent physically. The answer to this shall, however, be provided in a succeeding section using plots of the stream function, but first, we shall discuss how to find $Ra_{c}$ .

### 4.5. Expression for the Critical Rayleigh Number

The substitution of Equation (62) in the Rayleigh number expression (52) gives us:

(63) $$\begin{array}{c} Ra\left(n,\;S_{o}\right)=\frac{{\left[{\left(\delta \left(a_{2}-2\pi \right)+n\pi \right)}^{2}/S_{o}^{2}+\pi^{2}\right]}^{2}}{{\left(\delta \left(a_{2}-2\pi \right)+n\pi \right)}^{2}/S_{o}^{2}}\times \\ \left[\Lambda \left[{\left(\delta \left(a_{2}-2\pi \right)+n\pi \right)}^{2}/S_{o}^{2}+\pi^{2}\right]+\sigma^{2}\right]\left[1+\frac{H}{{\left(\delta \left(a_{2}-2\pi \right)+n\pi \right)}^{2}/S_{o}^{2}+\pi^{2}+H\gamma }\right]. \end{array}$$

In finding the critical Rayleigh number, $Ra_{c}$ , after which entropy generates in the system, for each value of $S_{o}$ we found that n=1 is not necessarily the value which yields $Ra_{c}$ . The value n=1 yields the minimum Rayleigh number until the aspect ratio crosses the threshold value, $S_{o}=2.17$ in CE . At $S_{o}=2.17$ , ${\left(Ra_{c}\right)}_{n=1}={\left(Ra_{c}\right)}_{n=2}$ , after which n=2 yields $Ra_{c}$ . Similarly after $S_{o}=3.50$ , n=3 in CE results minimum $Ra_{c}$ .

**Figure 2:**
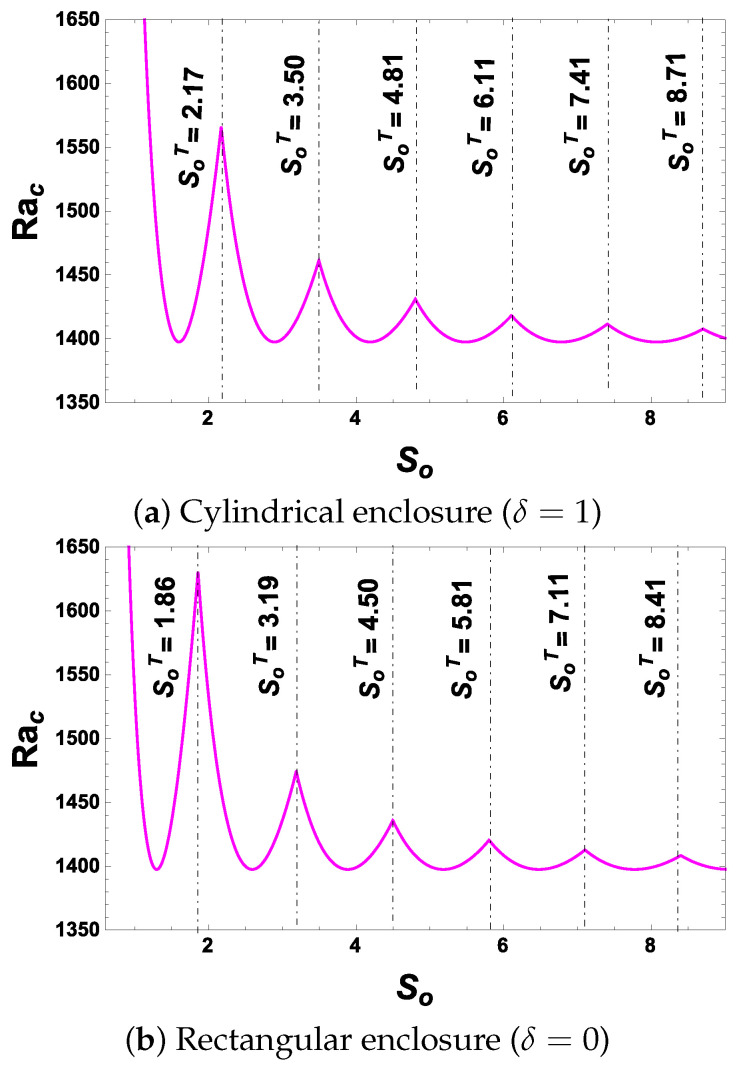
Plot of the critical Rayleigh number, $Ra_{c}$ , versus the aspect ratio, $S_{o}$ , for H=5,γ=18.7306,Λ=1.37655 and $\sigma^{2}=10$ values. $S_{o}^{T}$ refer to values of $S_{o}$ at intersection points.

This observation is graphically shown in Figure 2. For different ranges of $S_{o}$ , ascending integer values of *n* produces $Ra_{c}$ . The intersection points of piecewise continuous curves shown in Figure 2 actually represent the points of increase in the value of *n* (we have as yet not confirmed that *n* is the number of cells manifesting). To analyse this information from the physics point of view, we shall plot the stream function in the next subsection.

### 4.6. Streamlines and Physical Interpretation of n and a

Introducing velocity components *U* and *V* in terms of the stream function Ψ(S,Y) , we obtain

(64) $$U=-\frac{S_{o}\left[\delta \left(1-S\right)+S\right]}{S}\frac{\partial \Psi }{\partial Y},\;\;\;\;V=\frac{\left[\delta (1-S)+S\right]}{S}\frac{\partial \Psi }{\partial S}.$$

The velocity components *U* and *V* satisfy the continuity Equation (18). We have an explicit solution for V(S,Y) , and hence, we use this in obtaining an expression for the stream function to obtain

(65) $$\Psi =A_{0}\sin\left[\pi Y\right]\int \frac{S^{\frac{3-\delta }{2}}}{\delta (1-S)+S}J_{\frac{\delta -1}{2}}\left[a_{n}S\right]dS.$$

**Figure 3:**
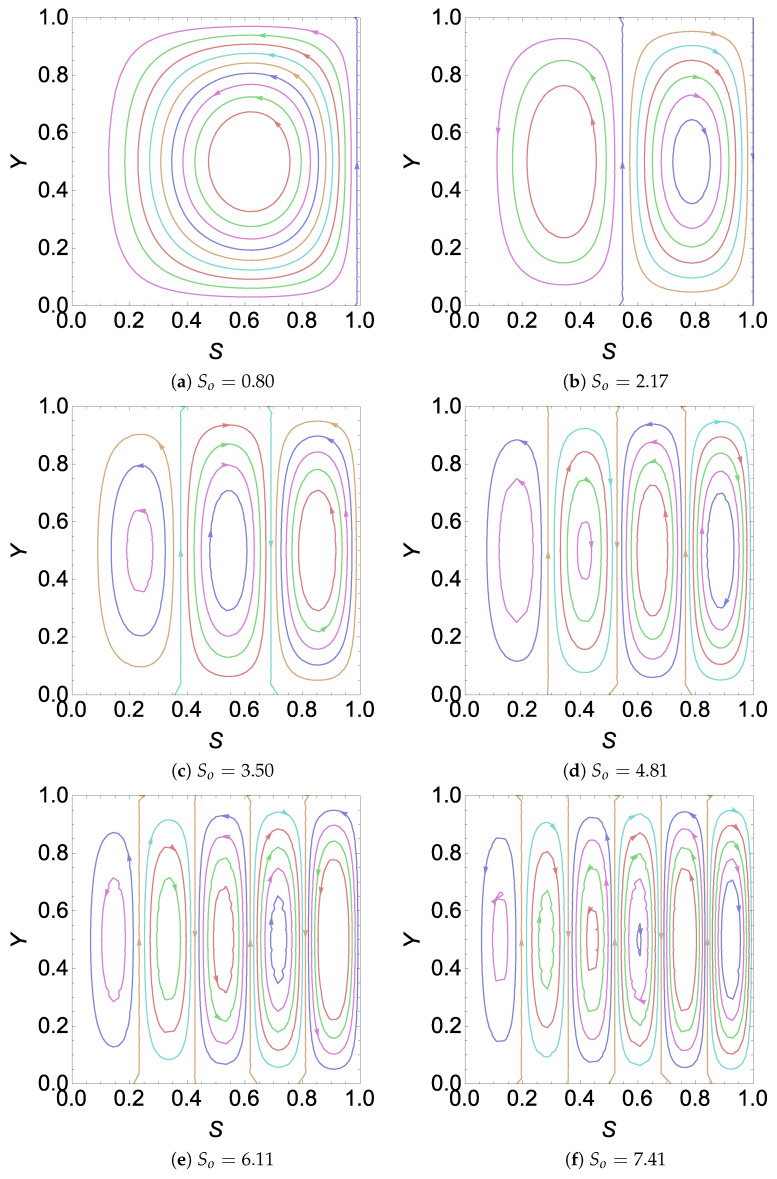
Plot of streamlines, Ψ , for different values of $S_{o}$ with H=5,γ=18.7306,Λ=1.37655 and $\sigma^{2}=10$ in a CE .

The integral constant in Equation (65) is zero since the boundary is a streamline. In Figure 3, we have plotted the stream function for a CE by considering one representative value of $S_{o}$ between two intersecting points in Figure 2a.

**Figure 4:**
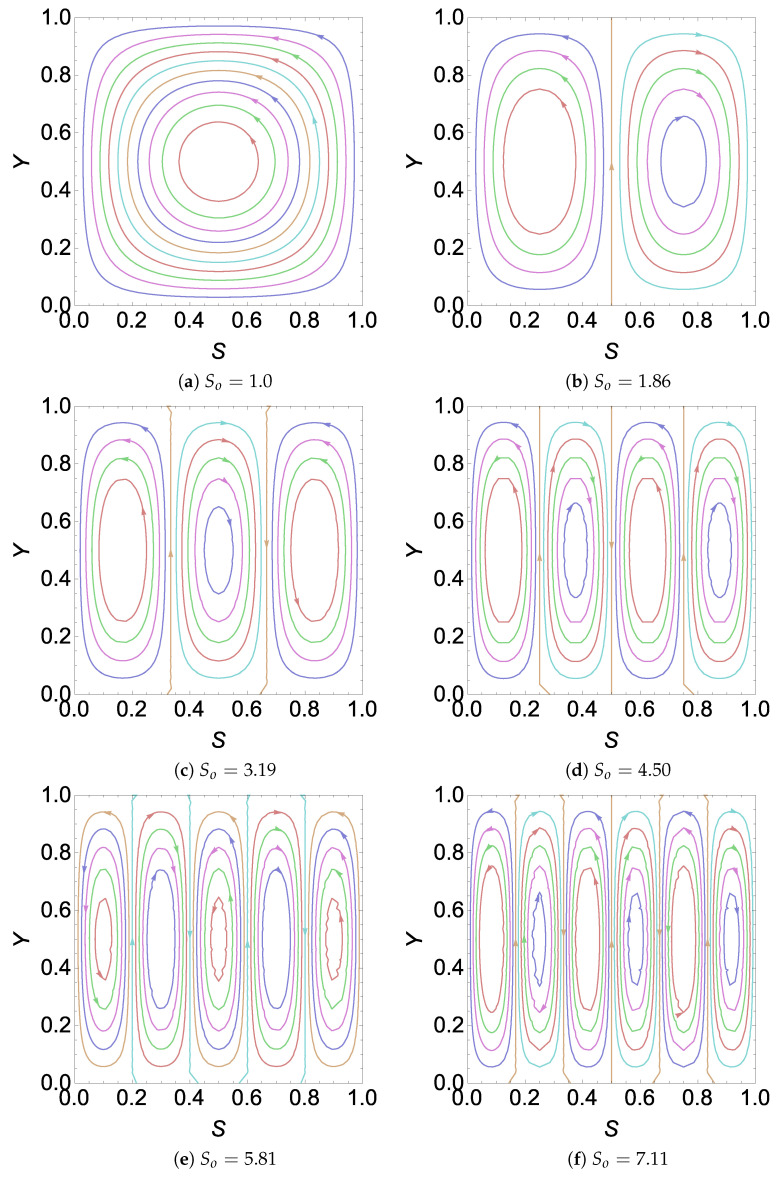
Plot of Ψ for different values of $S_{o}$ with H=5,γ=18.7306,Λ=1.37655 and $\sigma^{2}=10$ in a RE .

Now, on observing Figure 2a and Figure 3 together, we find that intersection points in Figure 2 represent the points at which the number of cells increases. Quite obviously, we may now infer that *n* must represent the number of cells for a given $S_{o}$ . Similar arguments can be made for RE by observing Figure 2b and Figure 4. From this observation in Figure 2, Figure 3 and Figure 4, we can find sufficient information to obtain an explicit expression for the number of cells in the cases of CE and RE . The details are given in the next section.

### 4.7. Explicit Expression for the Number of Cells in CE and RE

The intersection points of different modes (different *n*) in Figure 2 can be obtained by equating the critical Rayleigh number expressions of succeeding participating curves at their intersecting points. We thus take:

(66) $$Ra\left(n,\;S_{o}\right)=Ra\left(n+1,\;S_{o}\right),$$

where $S_{o}$ can now be only an intersection point. Now, using Equation (63) in Equation (66), we obtain an explicit expression for *n* as a function of $S_{o}$ and other parameters appearing in the problem, in the form:

(67) $$n=\frac{-\pi +\sqrt{\pi^{2}+4P}}{2\pi }+\left[2-\frac{a_{2}}{\pi }\right]\delta ,$$

where *P* is the real and positive root (only one, in fact) of the fifth-degree polynomial given by

(68) $$\frac{q_{4}}{2}\left(\pi^{2}+q_{3}\;S_{o}^{2}\right)S_{o}^{8}+q_{4}\;S_{o}^{8}x+\left(q_{5}\;S_{o}^{2}+q_{6}\;S_{o}^{6}\right)x^{2}+q_{7}\;S_{o}^{2}x^{3}+q_{8}x^{4}+2\Lambda x^{5}.$$

The various quantities in the above polynomial are given by:

(69) $$\begin{array}{c} q_{4}=-2\pi^{4}q_{1}\;q_{2},\\ q_{5}=\pi^{4}q_{3}\Lambda +q3\pi^{2}\left[q_{2}+\Lambda \left(q_{1}+2\pi^{2}\right)\right]S_{o}^{2},\\ q_{6}=\pi^{2}\left[q_{3}\sigma^{2}-q_{1}\left(\pi^{2}\Lambda +q_{2}\right)\right]+\left(q_{1}+\pi^{2}\right)\left[q_{3}\left(2\pi^{2}\Lambda +q_{2}\right)-\pi^{2}q_{2}\right],\\ q_{7}=4\pi^{2}\Lambda q_{3}+2q_{3}\left[\Lambda \left(q_{1}+2\pi^{2}\right)+q_{2}\right]S_{o}^{2},\\ q_{8}=\pi^{2}\Lambda +\left[\Lambda \left(q_{1}+2\pi^{2}\right)+q_{2}+3\Lambda q_{3}\right)S_{o}^{2}. \end{array}$$

The ceiling value of *n* represents the number of cells in the case of BBC with LTNE assumption. The ceil function returns the maximum integer value. The expression of *n* in the limiting cases of the present study is documented in Table 5.

The various quantities in Table 5 are given by

(70) $$\begin{array}{c} q_{9}={\left[-1-9\left(1+3\;S_{o}^{2}-3\;S_{o}^{4}\right)\;S_{o}^{2}+i\;6\sqrt{3(1+9\;S_{o}^{2}+27\;S_{o}^{4})}\;S_{o}^{3}\right]}^{\frac{1}{3}},\;i=\sqrt{-1},\\ q_{10}=1+3\;S_{o}^{2},\;q_{11}=\frac{1}{3}\left\{-2q_{16}+3q_{3}^{2}S_{o}^{4}+{\left(\frac{2}{q_{17}}\right)}^{\frac{1}{3}}\left[q_{16}^{2}-12\pi^{6}q_{1}S_{o}^{6}\right]\right\},\\ q_{12}=\frac{1}{3}{\left(\frac{q_{17}}{2}\right)}^{\frac{1}{3}},\;q_{13}=\frac{1}{3}\left\{2\left(-2q_{16}+3q_{3}^{2}S_{o}^{4}\right)-{\left(\frac{2}{q_{17}}\right)}^{\frac{1}{3}}\left[q_{16}^{2}-12\pi^{6}q_{1}S_{o}^{6}\right]\right\},\\ q_{14}=2\left[\left(q_{3}^{2}-2\pi^{2}q_{3}+\pi^{4}\right)\left(q_{1}-q_{3}\right)+\pi^{4}q_{1}\right]S_{o}^{6}+2\pi^{2}q_{3}^{2}S_{o}^{4},\\ q_{14}=2\left[\left(q_{3}^{2}-2\pi^{2}q_{3}+\pi^{4}\right)\left(q_{1}-q_{3}\right)+\pi^{4}q_{1}\right]S_{o}^{6}+2\pi^{2}q_{3}^{2}S_{o}^{4},\\ q_{15}=2\sqrt{q_{13}-q_{12}+\frac{q_{14}}{\sqrt{q_{11}+q_{12}}}},\;q_{16}=\left[q_{1}q_{3}+2\pi^{2}\left(q_{3}-q_{1}\right)-\pi^{4}\right]S_{o}^{4}+\pi^{2}q_{3}S_{o}^{2},\\ q_{17}=2q_{16}^{3}+36\pi^{4}q_{16}q_{1}\left[2q_{18}+q_{3}S_{o}^{2}\right]S_{o}^{6}+\sqrt{q_{19}}+108\pi^{4}q_{1}\left[\pi^{4}q_{1}S_{o}^{2}-q_{18}q_{3}^{2}\right]S_{o}^{10},\\ q_{18}=\pi^{2}+q_{3}S_{o}^{2}, \end{array}$$

(71) $$\begin{array}{c} q_{19}=4{\left[q_{16}^{3}+18\pi^{4}q_{1}q_{16}\left[2q_{18}+q_{3}S_{o}^{2}\right]S_{o}^{6}+54\pi^{4}q_{1}\left(\pi^{4}q_{1}S_{o}^{2}-q_{3}^{2}q_{18}\right)S_{o}^{10}\right]}^{2}\\ \;-4{\left[q_{16}^{2}-12\pi^{6}q_{1}S_{o}^{6}\right]}^{3},\\ q_{20}=\pi^{2}\Lambda +\left(3\pi^{2}\Lambda +\sigma^{2}\right)S_{o}^{2},\\ q_{21}=2\Lambda^{3}\pi^{6}\left(-1-9S_{o}^{2}-27S_{o}^{4}+27S_{o}^{6}\right)+6\pi^{4}\Lambda^{2}\sigma^{2}\left(-1-6S_{o}^{2}+9S_{o}^{4}\right)S_{o}^{2},\\ \;-6\pi^{2}\Lambda \sigma^{4}q_{10}S_{o}^{4}-2\sigma^{6}S_{o}^{6},\\ q_{22}={\left[q_{21}+\sqrt{-4q_{20}^{6}+q_{21}^{2}}\right]}^{\frac{1}{3}},\;q_{23}=\delta \left[2-\frac{a_{2}}{\pi }\right]. \end{array}$$

We note here that the quantity $\left[q_{10}\left(1-\frac{q_{10}}{q_{9}}\right)-q_{9}\right]$ appearing in Table 5 for the case of RBC evaluates to a real value with a very small imaginary part even though $q_{9}$ is a complex quantity.

Having obtained the expression for the number of cells in all possible cases, in the next section, we shall find the physical meaning of the parameter m .

### 4.8. Physical Interpretation of the Parameter m

To analyse the parameter m , we refer to Table 6, for water and four different water-saturated porous media considered wherein number of cells that shall appear for a given value of $S_{o}$ is documented in the cases of CE and RE . The corresponding cell sizes are calculated, and the same is recorded in Table 7.

From the tabulated values, we found that for small $S_{o}$ , the size of the cells is not the same in CE and RE . However, for large values of $S_{o}$ , these are the same for CE and RE . This means that the boundary effects are negligible at large $S_{o}$ , and we observe a uniformity in the cells. Hence, in this situation, the concept of wave number applies. This result is true for BBC with LTNE assumption and also in all its limiting cases.

Now recalling the definition of $m=\frac{a}{S_{o}}$ , we may write

(72) $$m_{c}=\frac{\delta (a_{2}-2\pi )+n\pi }{S_{o}}.$$

**Figure 5:**
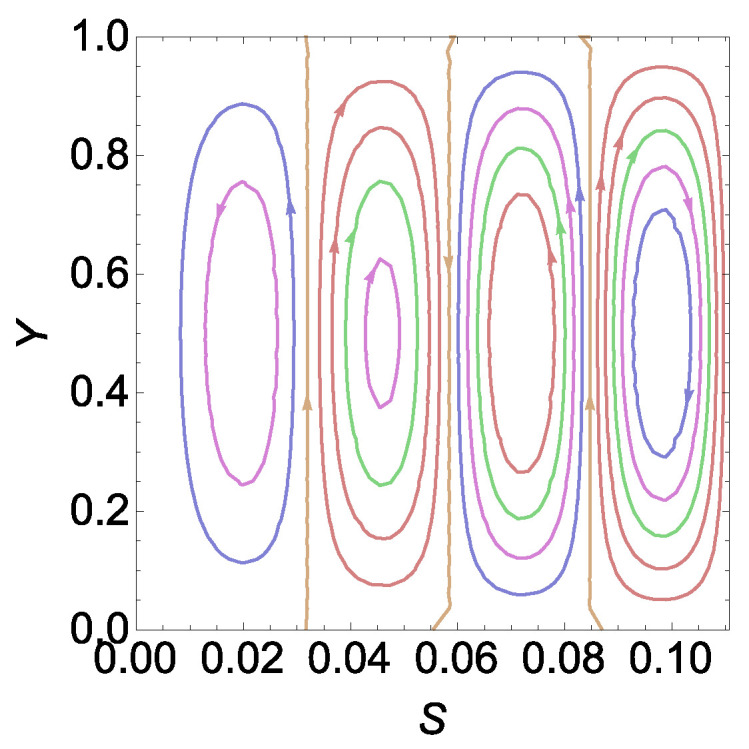
Plot of Ψ in the region $\left[0,\;\frac{a_{2}+2\pi }{a_{c}}\right]$ in a CE .

**Figure 6:**
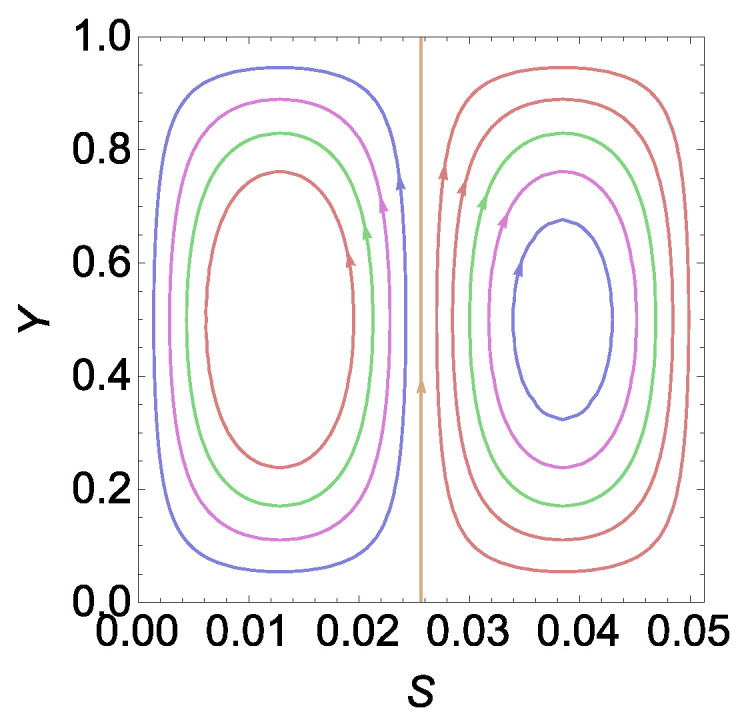
Plot of Ψ in the region $\left[0,\;\frac{2\pi }{a_{c}}\right]$ in a RE .

Here, *n* may be replaced by the expression of the number of cells given by Equation (67) for BBC – LTNE , and similarly, the expression of *n* of all limiting cases of BBC – LTNE documented in Table 5 can be used. Intuition tells us that the parameter m represents the wave number. To have confidence in this concept, we computed the value of $m_{c}=\frac{\delta (a_{2}-2\pi )+n\pi }{S_{o}}$ for $S_{o}$ in the range [10:100] for CE and RE . The computations reveal that in both CE and RE cases, the value of $m_{c}$ converges to the classical wave number of $\frac{\pi }{\sqrt{2}}$ . Hence, from now on, we shall refer to m as the wave number. Next, let us consider the stream function, Ψ , in the range $\left[0,\;\frac{\delta a_{2}+2\pi }{a_{c}}\right]$ in Figure 5 and Figure 6 for CE and RE .

For CE , we have chosen the range $\left[0,\;\frac{a_{2}+2\pi }{a_{c}}\right]$ due to the existence of the stagnant region in the first cell compared to all other cells. From Figure 5, it is clear that the range $\left[0,\;\frac{a_{2}}{a_{c}}\right]$ represents the initial wavelength, including the stagnant region, and the range $\left[\frac{a_{2}}{a_{c}},\;\frac{2\pi }{a_{c}}\right]$ represents the actual wavelength. In Figure 6, we shall see one wavelength or two counter-rotating cells of RE .

Now, some general observations made from the results of the study for any large $S_{o}$ are:

1.
$n^{RBC}<n^{BBC/DBC},\;\;m_{c}^{RBC}<m_{c}^{BBC/DBC}$ ,
2.
$n^{BBC}<n^{DBC},\;\;m_{c}^{BBC}<m_{c}^{DBC}$ ,
3.
$n^{LTNE}>n^{LTE},\;\;m_{c}^{LTNE}>m_{c}^{LTE}$ ,
4.
$n^{d=0.02m}>n^{d=0.03m},\;\;m_{c}^{d=0.02m}>m_{c}^{d=0.03m}$ .

Additional observations made from the tabulated values of Table 6 and Table 7 are as follows. In the case of LTE , the cell size depends on the permeability of the porous medium and not on its thermophysical properties. Hence, we observe that the cell size is the same for DBC−LTE involving the three porous materials considered. This result is also true for BBC−LTE . However, in the case of the corresponding problem under the LTNE assumption, we note that the cell size depends on not only the permeability but also the thermophysical properties of the porous medium. In that way, the results of BBC and DBC are different in so far as cell size is concerned when we consider the LTNE assumption. Another point to be observed is that the porous material with the largest thermal conductivity supports a smaller cell size.

Having obtained the expressions for the number of cells in different problems, we have recorded expressions of $Ra_{c}$ and *n* of all the problems (including limiting cases) in Table 8.

The quantities mentioned in Table 8 are present in Equations (33), (69), (70) and (71). Having so far discussed the results of the present paper, in the next section we draw some major conclusions of the study.

## 5. Conclusions

Two-dimensional analyses of Brinkman–Bénard convection of Newtonian liquid in cylindrical and rectangular geometries are made using the two-temperature model. In the present model, ten individual problems are unified into a single problem:

1.
BBC with LTNE in CE and RE ;
2.
BBC with LTE in CE and RE ;
3.
RBC in CE and RE ;
4.
BBC with LTE in CE and RE ;
5.
DBC with LTE in CE and RE .

One of the important results is that given the value of the aspect ratio and parameters’ values, we can exactly predict how many cells manifest (in all 10 possible cases). This is without seeking recourse to streamline plots. A number of cells seen in RBC are found to be less when compared with the corresponding problems in a porous medium, i.e., $n^{RBC}<n^{BBC}<n^{DBC}$ . This result is true in both LTNE and LTE and also for CE and RE . On comparing corresponding problems involving LTNE and LTE problems, we may write: $n^{LTNE}>n^{LTE}$ . For large aspect ratio, $S_{o}$ , the value of the wave number converges to $\frac{\pi }{\sqrt{2}}$ in both CE and RE , i.e., the vertical boundaries effect becomes negligible at large $S_{o}$ . This result coincides with the well-known result of a classical RBC problem, which validates the present model. However, the number of cells that manifest may not be the same for the corresponding problems in CE and RE , even for the case of $S_{o}\gg 1$ .

Work is in progress to consider the experimental boundary conditions similar to the idealistic boundary conditions considered in this paper. Liang et al. [33] experimentally observed the circular roll patterns in a CE bounded by rigid boundaries. This justifies the consideration of circular roll patterns in the present analysis in the case of CE . At this stage, the exact comparison of the present model with the experimental works is not possible as the present model considers the idealistic boundary conditions. The present study provides a qualitative picture of RBC in 10 different enclosures. The work on realistic boundary conditions is in progress. 

## Figures and Tables

**Table 1:** The values of parameters (Pr,α,γ,Λ) for water- GF , water-Sand, water- GB and water- AF .

	Pr	α	γ	Λ
water- GF	5.3393	0.9904	18.7306	1.37655
water-Sand	3.0336	0.6603	2.2703	5.6568
water- GB	3.0337	0.3177	0.5838	5.6568
water- AF	3.0337	0.0017	0.0030	5.6568

**Table 2:** The values of parameters *H* and $\sigma^{2}$ for water- GF , water-Sand, water- GB and water- AF .

	d(m)	*H*	$K(m^{-2})$	$\sigma^{2}$
water- GF	0.02	[0.5111:2.0446]	[657.28:10516.5]	[0.0038:0.0608]
	0.03	[599.37:5993.7]		[0.0085:0.1369]
water-Sand,	0.02	[2.4164:12.0824]	[0.2777:6.9444]	[5.7600:144.00]
water- GB ,		[2.5713:12.8867]		
water- AF		[2.5713:12.8867]		
water-Sand,	0.03	[1.0148:5.0741]	[340.556:3405.56]	[12.960:324.00]
water- GB ,		[1.0708:5.3543]		
water- AF		[1.0708:5.3543]		

**Table 3:** Asymptotic expressions of Ra in BBC and DBC cases with LTE assumption and with $m=\frac{a}{S_{o}}$ (applicable to both CE and RE ).

Cases	Ra [2]
BBC	$\frac{{\left(m^{2}+\pi^{2}\right)}^{2}}{m^{2}}\left[\Lambda \left(m^{2}+\pi^{2}\right)+\sigma^{2}\right]$
DBC $\left(\sigma^{2}\gg 1\right)$	$\frac{{\left(m^{2}+\pi^{2}\right)}^{2}}{m^{2}}$

**Table 4:** First seven roots of the constraint $J_{\frac{\delta +1}{2}}\left[a_{n}\right]=0$ in the cases of CE and RE .

*n*	0	1	2	3	4	5	6
$a_{n}\left(\delta =1\right)$	3.83171	7.01559	10.1735	13.3237	16.4706	19.6159	22.7601
$a_{n}\left(\delta =0\right)$	0	π	2π	3π	4π	5π	6π

**Table 5:** Expression for the number of cells in the limiting cases of BBC - LTNE .

	Parameters	Number of Cells
DBC (LTNE)	σ≫1	$n=\frac{1}{2\pi }\left[-\pi +\sqrt{\pi^{2}+2\sqrt{q_{11}+q_{12}}-2q_{3}S_{o}^{2}+q_{15}}\right]+q_{23}$
RBC	$\sigma^{2}=0$ and Λ=1	$n=\frac{-1}{2}+\sqrt{\frac{1}{4}-\frac{1}{6}\left[q_{10}\left(1-\frac{q_{10}}{q_{9}}\right)-q_{9}\right]}+q_{23}$
BBC (LTE)	H→0 or H→∞	$\frac{1}{2}\left[-1+\sqrt{1-\frac{2}{3\Lambda \pi^{2}}\left(q_{20}-\frac{2^{\frac{2}{3}}q_{20}^{2}+q_{22}^{2}}{2^{\frac{1}{3}}q_{22}}\right)}\right]+q_{23}$
DBC (LTE)	σ≫1 , H→0 or H→∞	$\frac{1}{2}\left[\sqrt{1+4S_{o}^{2}}-1\right]+q_{23}$

**Table 6:** Ranges of $S_{o}$ at which the number of cells increases in different water-saturated porous media with H=5.0 (in the case of LTNE ) and $H=10^{4}$ (in the case of LTE except for AF for which we need to take $H=10^{7}$ ), and d=0.03 m (in the case of BBC ) (The values in bold are the threshold values referred to as $S_{o}^{T}$ in Figure 2.

**Water-** pm	**Cases**	**Number of Cells (Cylindrical Enclosure)**							
		**1**	**2**	**3**	**4**	**5**	**6**	**7**	**8**
water- GF	BBC-LTNE	[0.68, 1.98)	[1.98, 3.19)	[3.19, 4.39)	[4.39, 5.58)	[5.58, 6.76)	[6.76, 7.95)	[7.95, 9.13)	[9.13, 10.32)
	BBC - LTE	[0.68, 1.99)	[1.99, 3.20)	[3.20, 4.40)	[4.40, 5.59)	[5.59, 6.78)	[6.78, 7.97)	[7.97, 9.15)	[9.15, 10.34)
water	RBC	[0.82, 2.37)	[2.37, 3.82)	[3.82, 5.25)	[5.25, 6.67)	[6.67, 8.09)	[8.09, 9.51)	[9.51, 10.92)	[10.92, 12.34)
water-sand	DBC-LTNE	[0.52, 1.59)	[1.59, 2.57)	[2.57, 3.54)	[3.54, 4.50)	[4.50, 5.46)	[5.46, 6.42)	[6.42, 7.38)	[7.38, 8.33)
	DBC-LTE	[0.54, 1.66)	[1.66, 2.69)	[2.69, 3.70)	[3.70, 4.71)	[4.71, 5.72)	[5.72, 6.72)	[6.72, 7.72)	[7.72, 8.72)
water- GB	DBC-LTNE	[0.5, 1.53)	[1.53, 2.48)	[2.48, 3.42)	[3.42, 4.34)	[4.34, 5.27)	[5.27, 6.20)	[6.20, 7.12)	[7.12, 8.04)
	DBC-LTE	[0.54, 1.66)	[1.66, 2.69)	[2.69, 3.70)	[3.70, 4.71)	[4.71, 5.72)	[5.72, 6.72)	[6.72, 7.72)	[7.72, 8.72)
water- AF	DBC-LTNE	[0.49, 1.50)	[1.50, 2.43)	[2.43, 3.34)	(3.34, 4.25)	[4.25, 5.16)	[5.16, 6.07)	[6.07, 6.97)	[6.97, 7.88)
	DBC-LTE	[0.54, 1.66)	[1.66, 2.69)	[2.69, 3.70)	[3.70, 4.71)	[4.71, 5.72)	[5.72, 6.72)	[6.72, 7.72)	[7.72, 8.72)
**Water**- pm	**Cases**	**Number of Cells (Rectangular Enclosure)**							
		**1**	**2**	**3**	**4**	**5**	**6**	**7**	**8**
water- GF	BBC-LTNE	(0, 1.70)	[1.70, 2.91)	[2.91, 4.11)	[4.11, 5.30)	[5.30, 6.49)	[6.49, 7.67)	[7.67, 8.86)	[8.86, 10.04)
	BBC - LTE	(0, 1.70)	[1.70, 2.92)	[2.92, 4.12)	[4.12, 5.31)	[5.31, 6.50)	[6.50, 7.69)	[7.69, 8.88)	[8.88, 10.06)
water	RBC	(0, 2.03)	[2.03, 3.48)	[3.48, 4.92)	[4.92, 6.34)	[6.34, 7.76)	[7.76, 9.18)	[9.18, 10.59)	[10.59, 12.01)
water-sand	DBC-LTNE	(0, 1.36)	[1.36, 2.34)	[2.34, 3.31)	[3.31, 4.28)	[4.28, 5.24)	[5.24, 6.20)	[6.20, 7.15)	[7.15, 8.11)
	DBC-LTE	(0, 1.42)	[1.42, 2.45)	[2.45, 3.47)	[3.47, 4.48)	[4.48, 5.48)	[5.48, 6.49)	[6.49, 7.49)	[7.49, 8.49)
water- GB	DBC-LTNE	(0, 1.31)	[1.31, 2.26)	[2.26, 3.20)	[3.20, 4.13)	[4.13, 5.06)	[5.06, 5.98)	[5.98, 6.91)	[6.91, 7.83)
	DBC-LTE	(0, 1.42)	[1.42, 2.45)	[2.45, 3.47)	[3.47, 4.48)	[4.48, 5.48)	[5.48, 6.49)	[6.49, 7.49)	[7.49, 8.49)
water- AF	DBC-LTNE	(0, 1.28)	[1.28, 2.22)	[2.22, 3.13)	[3.13, 4.04)	[4.04, 4.95)	[4.95, 5.86)	[5.86, 6.76)	[6.76, 7.66)
	DBC-LTE	(0, 1.42)	[1.42, 2.45)	[2.45, 3.47)	[3.47, 4.48)	[4.48, 5.48)	[5.48, 6.49)	[6.49, 7.49)	[7.49, 8.49)

**Table 7:** Cell-sizes of first 8 cells in different water-saturated porous media with H=5.0 (in the case of LTNE ) and $H=10^{4}$ (in the case of LTE except for AF for which we need to take $H=10^{7}$ ), and d=0.03 m (in the case of BBC ).

**Water-** pm	**Cases**	**Cell Aspect Ratios (Cylindrical Enclosure)**							
		**1**	**2**	**3**	**4**	**5**	**6**	**7**	**8**
water- GF	BBC-LTNE(d=0.003m)	1.30	1.21	1.20	1.19	1.18	1.19	1.18	1.19
	BBC-LTE(d=0.003m)	1.31	1.21	1.20	1.19	1.19	1.19	1.18	1.19
water	RBC	1.55	1.45	1.43	1.42	1.42	1.42	1.41	1.42
water-sand	DBC-LTNE	1.07	0.98	0.97	0.96	0.96	0.96	0.96	0.95
	DBC-LTE	1.12	1.03	1.01	1.01	1.01	1.00	1.00	1.00
water- GB	DBC-LTNE	1.03	0.95	0.94	0.92	0.93	0.93	0.92	0.92
	DBC-LTE	1.12	1.03	1.01	1.01	1.01	1.00	1.00	1.00
water- AF	DBC-LTNE	1.01	0.93	0.91	0.91	0.91	0.91	0.90	0.91
	DBC-LTE	1.12	1.03	1.01	1.01	1.01	1.00	1.00	1.00
**Water**- pm	**Cases**	**Cell Aspect Ratios (Rectangular Enclosure)**							
		**1**	**2**	**3**	**4**	**5**	**6**	**7**	**8**
water- GF	BBC-LTNE(d=0.03m)	1.21	1.20	1.19	1.19	1.18	1.19	1.18	1.19
	BBC-LTE(d=0.03m)	1.22	1.20	1.19	1.19	1.19	1.19	1.18	1.19
water	RBC	1.45	1.43	1.42	1.42	1.41	1.42	1.41	1.42
water-sand	DBC-LTNE	0.98	0.97	0.97	0.96	0.96	0.95	0.96	0.96
	DBC-LTE	1.03	1.02	1.01	1.00	1.01	1.00	1.00	1.00
water- GB	DBC-LTNE	0.95	0.94	0.93	0.93	0.92	0.93	0.92	0.92
	DBC-LTE	1.03	1.02	1.01	1.00	1.01	1.00	1.00	1.00
water- AF	DBC-LTNE	0.94	0.91	0.91	0.91	0.91	0.90	0.90	0.91
	DBC-LTE	1.03	1.02	1.01	1.00	1.01	1.00	1.00	1.00

**Table 8:** Expressions of $Ra_{c}$ and *n* in different problems.

Cases	${\mathrm{Ra}}_{c}$	$n[S_{o}]$
BBC (LTNE)	$\frac{{\left[{\left(\delta \left(a_{2}-2\pi \right)+n\pi \right)}^{2}/S_{o}^{2}+\pi^{2}\right]}^{2}}{{\left(\delta \left(a_{2}-2\pi \right)+n\pi \right)}^{2}/S_{o}^{2}}\left[\Lambda \left[{\left(\delta \left(a_{2}-2\pi \right)+n\pi \right)}^{2}/S_{o}^{2}+\pi^{2}\right]+\sigma^{2}\right]\times$ $\left[1+\frac{H}{{\left(\delta \left(a_{2}-2\pi \right)+n\pi \right)}^{2}/S_{o}^{2}+\pi^{2}+H\gamma }\right]$	$\frac{-\pi +\sqrt{\pi^{2}+4Q}}{2\pi }+\delta \left[2-\frac{a_{2}}{\pi }\right]$
DBC (LTNE)	$\frac{{\left[{\left(\delta \left(a_{2}-2\pi \right)+n\pi \right)}^{2}/S_{o}^{2}+\pi^{2}\right]}^{2}}{{\left(\delta \left(a_{2}-2\pi \right)+n\pi \right)}^{2}/S_{o}^{2}}\times \left[1+\frac{H}{{\left(\delta \left(a_{2}-2\pi \right)+n\pi \right)}^{2}/S_{o}^{2}+\pi^{2}+H\gamma }\right]$	$\frac{-\pi +\sqrt{\pi^{2}+4Q}}{2\pi }+\delta \left[2-\frac{a_{2}}{\pi }\right]$
RBC	$\frac{{\left[{\left(\delta \left(a_{2}-2\pi \right)+n\pi \right)}^{2}/S_{o}^{2}+\pi^{2}\right]}^{3}}{{\left(\delta \left(a_{2}-2\pi \right)+n\pi \right)}^{2}/S_{o}^{2}}$	$n=\frac{-1}{2}+\sqrt{\frac{1}{4}-\frac{1}{6}\left[q_{10}\left(1-\frac{q_{10}}{q_{9}}\right)-q_{9}\right]}+\delta \left(2-\frac{a_{2}}{\pi }\right)$
BBC (LTE)	$\frac{{\left[{\left(\delta \left(a_{2}-2\pi \right)+n\pi \right)}^{2}/S_{o}^{2}+\pi^{2}\right]}^{2}}{{\left(\delta \left(a_{2}-2\pi \right)+n\pi \right)}^{2}/S_{o}^{2}}\left[\Lambda \left[{\left(\delta \left(a_{2}-2\pi \right)+n\pi \right)}^{2}/S_{o}^{2}+\pi^{2}\right]+\sigma^{2}\right]$	$\frac{1}{2}\left[-1+\sqrt{1-\frac{2}{3\Lambda \pi^{2}}\left(q_{20}-\frac{2^{\frac{2}{3}}q_{20}^{2}+q_{22}^{2}}{2^{\frac{1}{3}}q_{22}}\right)}\right]+\delta \left[2-\frac{a_{2}}{\pi }\right]$
DBC (LTE)	$\frac{{\left[{\left(\delta \left(a_{2}-2\pi \right)+n\pi \right)}^{2}/S_{o}^{2}+\pi^{2}\right]}^{2}}{{\left(\delta \left(a_{2}-2\pi \right)+n\pi \right)}^{2}/S_{o}^{2}}$	$\frac{1}{2}\left[\sqrt{1+4S_{o}^{2}}-1\right]+\delta \left[2-\frac{a_{2}}{\pi }\right]$

## Data Availability

No new data were created or analyzed in this study. Data sharing is not applicable to this article.

## Nomenclature

***Latin Symbols***	
*b*	horizontal dimension of the enclosure (m)
*c*	specific heat at constant pressure $\;(J\;{\mathrm{kg}}^{-1}K^{-1})$
*d*	height of the enclosure (m)
$d_{s}$	diameter of the solid spheres (porous materials) (m)
*g*	acceleration due to gravity $\;(ms^{-2})$
*H*	scaled inter-phase heat transfer coefficient
*h*	inter-phase heat transfer coefficient $\;(Wm^{-2}K^{-1})$
*k*	thermal conductivity $\;(Wm^{-1}K^{-1})$
*m*	wave number
*n*	modes/number of cells
*P*	dynamic pressure (Pa)
Pr	Prandtl number
$\vec{Q^{*}}$	velocity vector
(S,Y)	dimensionless form of horizontal and vertical axes
(s,y)	horizontal and vertical axes (m)
$S_{o}$	aspect ratio
Ra	porosity-modified thermal Rayleigh number
Re	Reynolds number
*T*	temperature (K)
*t*	time (s)
(u,v)	velocities $\;(ms^{-1})$
(U,V)	nondimensional velocities
***Greek symbols***	
α	thermal diffusivity ratio
β	thermal expansion coefficient $\;(K^{-1})$
χ	thermal diffusivity $\;(m^{2}s^{-1})$
δ	curvature parameter
Δ	difference in quantities
γ	porosity-weighted thermal conductivity ratio
K	permeability $\;(m^{2})$
Λ	Brinkman number
μ	dynamic coefficient of viscosity $\;(\mathrm{kg}/m^{-1}s^{-1})$
Φ,Θ	dimensionless solid and fluid temperatures
ϕ	porosity $\left(0<\epsilon \leq 1\right)$
Psi	stream function
ρ	density $\left(\mathrm{kg}/m^{3}\right)$
$\sigma^{2}$	porous parameter
τ	dimensionless time
***subscripts***	
*b*	basic state
*c*	critical
*e*	effective quantities
*f*	fluid
*s*	solid(porous medium)
0	reference value
′	perturbed state
***abbreviations***	
AF	aluminium foam
BBC	Brinkman-Bénard convection
CE	cylindrical enclosure
DBC	Darcy-Bénard convection
GB	glass balls
GF	glass fiber
LTE	local thermal equilibrium
LTNE	local thermal nonequilibrium
RBC	Rayleigh-Bénard convection
RE	rectangular enclosure
